# Prevalence and Socio-Behavioural Determinants of Periodontal Disease Among Adults in the Northern West Bank: A Cross-Sectional Study

**DOI:** 10.3390/dj14010053

**Published:** 2026-01-13

**Authors:** Sura Al-Hassan, Mazen Kazlak, Elham Kateeb

**Affiliations:** 1 Faculty of Public Health, Al-Quds University, Jerusalem 51000, Palestine; 2 Faculty of Medicine and Health Sciences, An-Najah National University, Nablus 00970, Palestine; mazen.kazlak@najah.edu; 3 Oral Health Research and Promotion Unit, Faculty of Dentistry, Al-Quds University, Jerusalem 51000, Palestine

**Keywords:** periodontal diseases, community periodontal index for treatment needs, adult oral health, oral hygiene practices, socio-behavioural determinants, regression

## Abstract

**Background & Objectives**: Periodontal disease (PD) is a common oral disease that affects the supporting structures of the teeth and is a leading cause of tooth loss worldwide. This study aimed to estimate the prevalence of PD among 9th-grade teachers in the northern West Bank and examine its association with key behavioral and socioeconomic factors. **Methods**: A cross-sectional study was conducted among 920 teachers selected through proportional stratified random sampling from governmental and private schools. Periodontal health was assessed using the WHO Community Periodontal Index for Treatment Needs (CPITN), and oral hygiene status was measured with the Simplified Oral Hygiene Index (S-OHI). A structured questionnaire was administered to collect data on socioeconomic status, oral hygiene practices, dietary habits, and smoking behaviours. Data was analysed using descriptive statistics, bivariate and multivariate logistic regression. **Results**: Only 11.8% of participants exhibited completely healthy gingiva, with the mean condition ranging between calculus and shallow pockets. Oral hygiene practices were the strongest predictors of periodontal outcomes: frequent tooth brushing (Adjusted Odds Ratio: AOR = 0.015), morning brushing (AOR = 0.015), and regular toothbrush replacement (AOR = 2.514) were protective. Higher red meat intake was negatively associated with periodontal health (AOR = 0.032), while frequent nut consumption was protective (AOR = 0.227). The number of cigarettes smoked per week was positively associated with PD (AOR = 1.085). **Conclusions**: PD is highly prevalent among Palestinian adults, with significant behavioural and lifestyle-related determinants. Targeted oral health interventions are urgently needed to improve adults’ oral health.

## 1. Introduction

Periodontal diseases (PDs) are among the most prevalent oral health diseases globally, representing a major public health concern [1,2] and a leading cause of tooth loss in adults [3,4]. Epidemiological data indicate that PDs affect between 20% and 50% of the global population, ranking as the sixth most common human disease [2,5]. Despite advances in preventive and therapeutic strategies, the burden of PDs has remained relatively unchanged over recent decades, with severe periodontitis affecting an estimated one billion adults worldwide by 2021, corresponding to an age-standardized prevalence of approximately 12.5% [6].

Marked disparities persist across regions and income levels. In high-income countries, the prevalence of PD remains substantial, primarily attributed to population ageing and the cumulative effects of chronic inflammation and systemic comorbidities such as diabetes and cardiovascular disease [7]. Conversely, low- and middle-income countries experience a disproportionate burden of moderate to severe forms, driven by limited access to oral healthcare services, inadequate preventive programs, lower oral health literacy, and increased exposure to behavioural risk factors including tobacco use, poor nutrition, and low socioeconomic status (SES) [8].

Clinically, PD encompasses a spectrum of chronic inflammatory disorders characterized by the destruction of the supporting tooth structures—the periodontal ligament and alveolar bone—ultimately leading to tooth mobility and loss if left untreated [4,5]. Gingivitis represents the initial, reversible stage of the disease, presenting as inflammation confined to the gingival tissues. Progression into periodontitis involves apical migration of the junctional epithelium, formation of periodontal pockets, and resorption of alveolar bone [5].

PD is primarily driven by modifiable behavioural factors, particularly oral hygiene practices, dietary habits, and smoking behaviors [9,10]. Insufficient brushing and inadequate interdental cleaning contribute to plaque buildup and microbial dysbiosis at the gingival margin, triggering inflammatory responses that can progress to attachment loss and alveolar bone resorption [9,10,11]. Unhealthy dietary patterns rich in refined sugars and saturated fats exacerbate both local and systemic inflammation. At the same time, an insufficient intake of fruits, vegetables, and antioxidants impairs immune defense and tissue repair [12,13]. Smoking further accelerates periodontal destruction by compromising immune responses, reducing gingival blood flow, and altering the subgingival microbiota. A clear dose–response relationship exists between tobacco use and disease severity, and partial recovery of periodontal parameters has been documented following cessation [14,15,16].

Evidence from the Eastern Mediterranean region underscores persistent gaps in oral health awareness and preventive behaviors, even among populations with higher educational attainment. A multicountry study among Arab dental students in Lebanon, Syria, and Tunisia reported modest mean scores on oral health behaviours and attitudes, with significantly lower scores among those who smoked or engaged in other adverse health behaviours, highlighting the influence of behavioural risk factors even within health-educated groups [17]. Locally, in Palestine, Kateeb et al. [18] found that pregnant women exhibited widespread gaps in oral health knowledge, inappropriate oral hygiene habits, and erroneous beliefs regarding dental care during pregnancy. This remains one of the few studies examining adults’ oral health behaviors in the country, emphasizing the need for broader population-based investigations and context-specific educational interventions. Collectively, such findings affirm that awareness and behavioural determinants are central to understanding and addressing the burden of PD across diverse adult populations. To summarize, multiple lifestyle-related oral health factors, particularly oral hygiene practices, dietary habits, and smoking behaviors, play a pivotal role in the onset and progression of PD by impairing host immune responses and amplifying the inflammatory destruction of periodontal tissues [19]. Recognizing the need to explore how these behavioural determinants manifest themselves in adults, the present study focuses on 9th-grade teachers as a population of particular interest. The selection of this group was purposeful, as this study is part of a broader research initiative conducted by the same team, complementing a previously published study involving 9th-grade students from the same schools [20]. Both studies employed a similar methodological framework, utilizing the same dental indices and standardized data collection procedures. This design facilitated a comprehensive analysis of periodontal health within the school environment. While the initial study focused on adolescent populations, the current investigation specifically examines their teachers, thereby offering an adult perspective on the socio-behavioral factors that influence PD. Investigating the teachers of these students, therefore, offers an opportunity to examine PD within an adult cohort directly connected to adolescents already shown to be at risk.

Teachers represent a socially influential segment of the community who can model and transmit oral health-related behaviors to younger generations. Their lifetime experiences with oral hygiene, dietary choices, and smoking, coupled with varying socioeconomic circumstances, make them an informative group for understanding community-level determinants of PD [21,22,23,24]. Furthermore, their occupational setting provides a feasible platform for implementing and evaluating workplace-based oral health promotion programs.

Given the scarcity of data on periodontal conditions among adults in Palestine, a critical gap remains in understanding the status of adult periodontal health and its behavioral correlates. This study, therefore, aimed to (1) determine the prevalence of PD among 9th-grade teachers in public and private schools across the northern governorates of the West Bank, and (2) assess the associations between PD and selected determinants, including socioeconomic status, oral hygiene practices, dietary habits, and smoking behaviours. The findings from this study are expected to inform the design of targeted, evidence-based interventions and oral health promotion strategies for adult populations in the region.

## 2. Methodology

### 2.1. Study Design and Setting

The study was conducted in accordance with the Declaration of Helsinki and was approved by the Ethics Committee of Al-Quds University (Ref. No. 13/24, approval date: 15 March 2024). This cross-sectional study was conducted in schools across the northern governorates of the West Bank, which include Jenin, Tulkarm, Nablus, Qalqilya, and Salfit. These governorates collectively have approximately 1.1 million people and encompass 933 schools. Of these, 813 are government-run, 38 are operated by the United Nations Relief and Works Agency for Palestine Refugees, and 82 are privately managed [25].

### 2.2. Sampling Process

A multilevel sampling framework was implemented to ensure that the selected group of 9th-grade teachers accurately represented the original 9th-grade teacher population across the northern West Bank governorates. The process began by identifying all schools with 9th-grade teachers in the governorates of Nablus, Jenin, and Tulkarm. These governorates were prioritized because they account for a significant portion of the teacher workforce and show considerable diversity in demographic, socioeconomic, and institutional characteristics.

**Figure 1:**
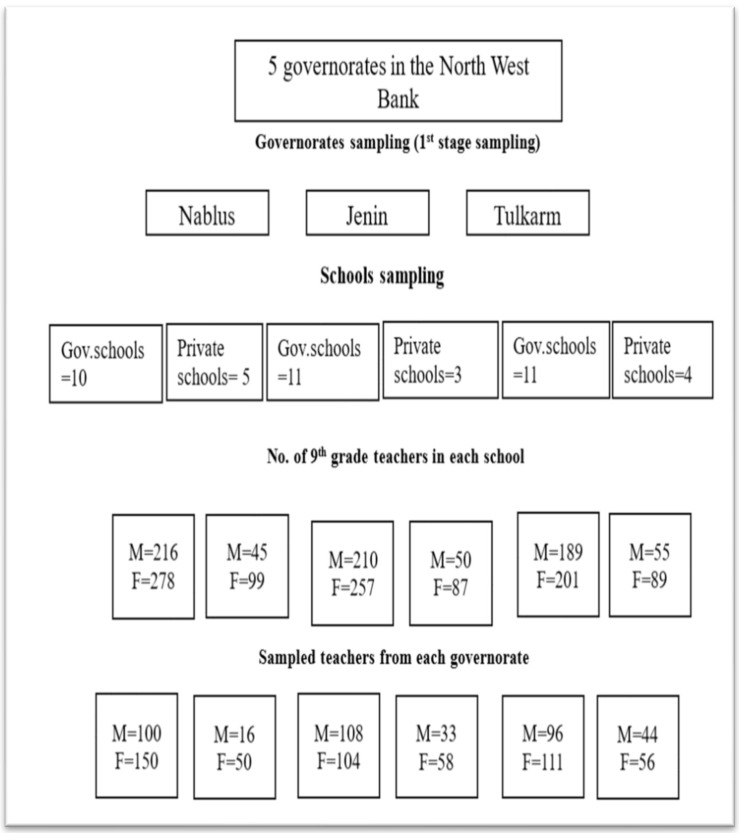
Flowchart of the sampling procedure across governorates, schools, and teachers.

In the first stage, a proportional stratification approach was utilized based on two key criteria: (1) school type (government or private) and (2) teacher gender (male or female). This stratification ensured that teachers included in this study had different educational systems and work environments. After obtaining a complete list of eligible teachers from the Palestinian Ministry of Education, the teachers were assigned to a stratum that corresponded to their proportion of the total teacher population. Teachers were then randomly selected from within each stratum, resulting in a final sample of 920 teachers. This sample was distributed in accordance with the actual teacher distribution across the three governorates. Figure 1 provides a schematic overview of the sampling process, illustrating the progression from the governorate level to the school level and finally to the teacher level.

### 2.3. Pilot Study

A preliminary pilot study was conducted to refine the research questionnaire and evaluate the practicality and feasibility of the study procedures before starting full-scale data collection [26,27]. The pilot involved approximately 10% of the intended sample [28], comprising 100 teachers who met the eligibility criteria and consented to participate in this study. This phase also provided an opportunity to assess the clarity of the questionnaire items, the feasibility of administering both the interview and clinical examination components, and the overall logistics of coordinating school visits.

The feedback from this pilot survey was thoroughly reviewed to identify any ambiguities or redundancies in the questionnaire. Based on this feedback, several questions were modified to enhance clarity and ensure that each one accurately reflected the intended behavioral or sociodemographic construct. Additionally, the research team took this opportunity to assess the flow of the clinical examination process and to confirm that data collection could be conducted efficiently within the school setting.

To establish the robustness of the instrument, several validity checks were conducted. Face validity was confirmed by ensuring that participants found the questions relevant and understandable [29]. Content validity was assessed by a panel of experts in periodontology, public health, and clinical research, who verified that the questionnaire comprehensively represented the study variables [30]. Additionally, an Exploratory Factor Analysis (EFA) using Principal Component Analysis was performed to evaluate construct validity. This was done after confirming that the data met the assumptions for factorability, as indicated by the Kaiser–Meyer–Olkin (KMO) measure and Bartlett’s Test of Sphericity.

While the pilot study provided valuable insights that improved the final instrument and procedures, its data were excluded from the main analysis to uphold methodological rigor.

### 2.4. Clinical Screening

This research employed the Community Periodontal Index for Treatment Needs (CPITN) to assess the prevalence of PD among 9th-grade teachers. CPITN is widely recognized as a dependable instrument for measuring PD prevalence and is often utilized to shape health policy and inform the development of PD management strategies [31,32,33]. Compared with other techniques that require comprehensive evaluations of clinical attachment loss, such as the Basic Periodontal Examination (BPE) [34], CPITN offers a more straightforward methodology. The functional attributes of CPITN have been extensively reviewed in existing literature, including comparisons to authoritative periodontal assessments and diagnostic standards. Studies have shown that its sensitivity, specificity, and both positive and negative predictive values are 58%, 80%, 87%, and 46.3%, respectively [35]. Furthermore, CPITN has proven to be effective in diagnosing both gingivitis and periodontitis [36]. In adults, CPITN employs scores of 0, 1, 2, 3, and 4 to evaluate gingivitis, with the following designations: Score 0 indicates healthy gingiva, Score 1 signifies gingival bleeding upon gentle probing, Score 2 reflects the presence of supra- and/or sub-gingival calculus, Score 3 denotes shallow pockets ranging from 4 to 5 mm, and Score 4 represents deep pockets of 6 mm or more. For this study, the designated index teeth examined were 17, 16, 11, 26, 27, 31, 36, 37, 46, and 47 [37].

This study also used the Simplified Oral Hygiene Index (S-OHI) [38] to assess the participants’ oral hygiene status. This index has been extensively utilised in oral health research [39,40]. This index comprises two components: the Debris Index–Simplified (DI-S), which represents the soft plaque index, and the Calculus Index–Simplified (CI-S), which represents the calcified plaque index. Debris Index–Simplified (DI-S)—Soft Plaque: Score 0: No soft debris or extrinsic stain, 1: Soft debris covering not more than one-third of the tooth surface, Score 2: Soft debris covering more than one-third but not more than two-thirds of the tooth surface, Score 3: Soft debris covering more than two-thirds of the tooth surface. While the Calculus Index–Simplified (CI-S)—Calcified Plaque have the following scores: Score 0: No calculus present, Score 1: Supragingival calculus covering not more than one-third of the tooth surface, Score 2: Supragingival calculus covering more than one-third but not more than two-thirds of the tooth surface, or isolated flecks of subgingival calculus around the cervical portion of the tooth, Score 3: Supragingival calculus covering more than two-thirds of the tooth surface, or a continuous heavy band of subgingival calculus around the cervical portion of the tooth. Six index teeth (16, 11, 26, 36, 31, and 46) were examined on specific surfaces (buccal for upper molars and lower incisors, lingual for lower molars, and labial for upper incisors). For each participant, DI-S and CI-S scores were calculated by summing the scores across the examined surfaces and dividing by the number of surfaces. The OHI-S score was obtained by adding the DI-S and CI-S scores, yielding a range from 0 to 6, with higher scores indicating poorer oral hygiene.

During the pilot phase of this study, inter-examiner reliability was evaluated using a two-way random-effects model of the Intraclass Correlation Coefficient (ICC), as described by Koo and Li [41]. A total of five trained data collectors conducted independent assessments of 20 teachers on five separate occasions. The results yielded ICC values of 0.79 for CPITN and 0.89 for S-OHI, both indicating strong inter-examiner reliability. The selection of the ICC model was based on the premise that the raters were randomly selected from a broader population of dentists practising in the West Bank and maintained consistent participation throughout the study, as supported by the works of Portney et al. [42] and Bruton et al. [43].

### 2.5. Questionnaire

The questionnaire utilized in this research was developed based on existing literature [44,45,46]. Its objective was to assess several PD determinants, including oral hygiene practices, dietary habits, and smoking behaviors. The original questionnaire was in English and later translated into Arabic. To ensure the translation’s accuracy, a back-translation process was implemented, where the Arabic version was converted back into English. This step was essential to verify the translation’s fidelity prior to administering the questionnaire to participants. The questionnaire comprised standardized, pretested, closed-ended items and underwent extensive evaluation of face and content validity by a group of three experts. The questionnaire was structured into five sections to address the major determinants associated with PD. Section 1 gathered information on the teachers’ demographics, including their gender and the type of school in which they work. Section 2 concentrated on family background, encompassing family size and financial status. Section 3 assessed oral hygiene practices, specifically looking into the frequency, duration, and techniques of tooth brushing. Section 4 examined dietary habits, focusing on daily consumption of nutritious versus non-nutritious foods. Lastly, Section 5 investigated smoking habits, measuring both the frequency of cigarette and waterpipe usage and the number of cigarettes smoked. The internal consistency of the questionnaire was evaluated using Cronbach’s alpha. Reliability analysis conducted on a sample of 100 cases yielded an alpha of 0.967 across 45 items, indicating outstanding internal consistency.

### 2.6. Data Collection Process

Following ethical approval from the Al-Quds committee (REF. 13/24), consent forms were sent to the participant, and data collection commenced upon receiving the approval.

Before initiating the fieldwork, the principal investigator trained five dentists to standardize both interviewing procedures and dental examination techniques. These training sessions were held repeatedly throughout the study period to maintain a consistent approach among all examiners.

Data collection consisted of two sequential components: a face-to-face interview followed by dental screening. Each teacher was interviewed individually in a private setting on school premises to ensure confidentiality and allow sufficient time to clarify any questions. The structured interview captured sociodemographic characteristics and self-reported behavioural determinants. Immediately after the interview, a trained dentist performed the dental assessment using CPITN and S-OHI criteria. All examinations were conducted under natural light, using disposable examination kits and WHO periodontal probes. Infection-control protocols were followed rigorously throughout the process.

The fieldwork spanned approximately two months, during which all eligible and consenting teachers were approached. Completed questionnaires and clinical forms were reviewed daily by the research team to ensure accuracy and completeness. Subsequently, all data were coded, anonymized, and stored in encrypted files accessible only to authorized members of the research team, in accordance with institutional confidentiality standards.

### 2.7. Data Management and Statistical Analysis

Statistical analysis was performed using IBM SPSS version 25.0.

To evaluate the CPITN, the highest recorded score within examined mouth regions was utilised as the representative score for each participant [44], and its frequency was subsequently calculated. Additionally, the mean score of the Simplified Oral Hygiene Index (S-OHI) was calculated. The S-OHI mean scores were classified according to Greene and Vermillion [38] as follows: good (0.0–1.2), fair (1.3–3.0), and poor (3.1–6.0).

Descriptive statistics, including frequencies and percentages, were used to describe all variables for the participants in the study. The distribution of CPITN categories across the examined mouth regions and participants was also thoroughly analyzed.

To identify the determinants that influence periodontal status among the participant teachers, logistic regression statistical analysis was selected due to the non-normal distribution of the original CPITN scores (the dependent variable) [47,48]. In turn, bivariate logistic regression for CPITN data was used (as the first step in analysis) because it is a commonly used and accepted approach in the international peer-reviewed literature [49]. Consequently, the CPITN scores were reclassified into a binary system: healthy gingiva (0) and diseased gingiva (1). This approach adhered to the WHO guidelines for epidemiological studies [44], and the binary reclassification system served the main study’s aim, which was to assess the determinants of PD. Furthermore, this binary system simplified the analysis and interpretation of the findings.

Before analysis, missing values were imputed. The K-nearest neighbour (K-NN) imputation method was employed, resulting in accurate data completion for each case [50]. Outliers were also identified using boxplots and metrics of skewness and kurtosis, and subsequently trimmed to exclude extreme cases.

Regarding multicollinearity, no independent variables had a Variance Inflation Factor (VIF) value below 5, and no tolerance value was less than 2.5, indicating no multicollinearity issues.

To prepare the variables for regression analysis, dummy coding was applied, which is a commonly used method for representing categorical variables [51]. Most variables in this study were recoded into binary indicators (0/1), with one category designated as the reference level (coded 0). However, some variables had three categories, such as the governorate variable. On the other hand, dietary habits were treated as scale variables because when dietary variables were transformed into categorical indicators, the statistical significance of the associations decreased, likely due to the loss of information and reduced statistical power. Retaining these variables as scale measures allowed us to preserve the variability in the data and enhance the sensitivity of the regression analysis.

Before model fitting, prespecified checks were performed to ensure sufficient cell sizes and an appropriate number of events per variable (EPV ≈ 5–10 per coefficient) [52,53].

To ensure the validity of the binary logistic regression model, the Box-Tidwell test was applied to verify linearity in the continuous predictors. This involved adding a product term between each predictor and its natural log transformation and examining the significance of this term. The results revealed a non-significant interaction term (B = −0.001, *p* = 0.789), confirming that the assumption of linearity in the logit was met.

Socio-behavioural factors, such as SES, oral hygiene practices, dietary habits, and smoking behaviours, were considered potential predictors for CPITN [54,55,56]. Based on the previous literature, only those variables that presented with a significance level of (*p* ≤ 0.05) in bivariate logistic regression were included in the multivariate analysis. In contrast, variables that showed no significant association in bivariate analyses (*p* ≤ 0.05) were excluded from the multivariate analysis [57].

The study employed a stepwise forward inclusion procedure for model development, beginning with an empty model. Predictors were added one by one based on their statistical significance, continuing until no further variables met the inclusion criteria. The final model retained only statistically significant predictors (*p*-value < 0.05). Adjusted odds ratios were calculated with their respective 95% confidence intervals.

The Hosmer–Lemeshow test was used to assess the model’s goodness-of-fit. The logistic regression model’s events-per-variable (EPV) ratio was determined by dividing the total number of outcome events (920) by the number of independent predictors (29). This results in an EPV of approximately 31.7, which is significantly above the recommended threshold of 10. This finding suggests that the risk of overfitting is minimal, as noted by Vittinghoff and McCulloch (2007) [58]. Such a strong EPV ratio indicates the model’s stability and the results’ generalizability [58].

## 3. Results

The distribution of periodontal conditions among 920 participant teachers demonstrates significant variation across different tooth regions. Notably, the lower right second molar region emerged as the healthiest area, showcasing the highest proportion of healthy gingiva at 72.7%. In contrast, the upper central incisor region exhibited the highest prevalence of gingival bleeding at 62.3%, indicating its vulnerability to bleeding.

**Figure A1:**
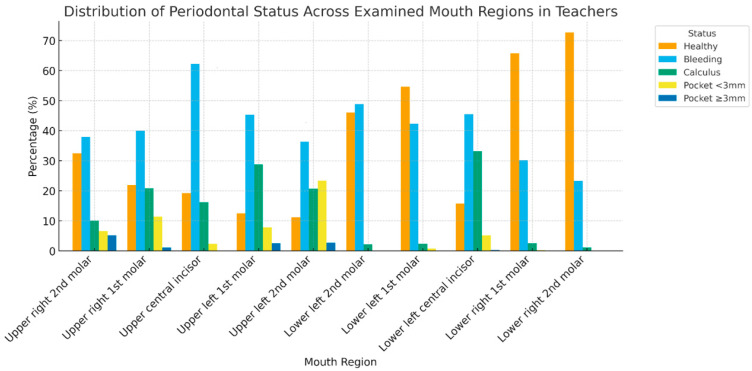
Distribution of Periodontal Status Across Examined Mouth Regions in the Participant Teachers.

Additionally, the lower left central incisor region displayed the highest rate of calculus deposition at 33.2%, reflecting a substantial accumulation of plaque and calculus in this region. Regarding periodontal pockets, the upper left second molar region presented the highest occurrence of shallow pockets (3–4 mm), affecting 23.4% of participants. In comparison, deeper pockets (≥6 mm) were most frequently observed in the upper right second molar region, with a prevalence of 5.2% (Table 1) (See Figure A1).

**Figure A2:**
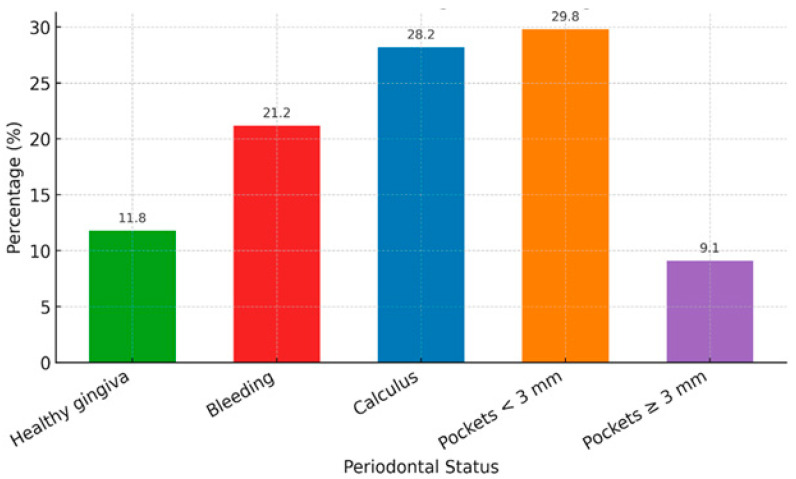
Distribution of (CPITN) Categories Among Teachers.

The CPITN distribution among the participant teachers shows that only about one in nine (11.8%) had completely healthy gingiva, while a fifth (21.2%) exhibited bleeding on probing. Calculus deposits were present in just over a quarter of participants (28.2%), and nearly three in ten (29.8%) had shallow periodontal pockets (3–4 mm). Deep pockets (≥6 mm), indicative of more advanced periodontal breakdown, were observed in 9.1% of the sample. The overall mean CPI score was 3.03 (SE = 0.0382; 95% CI = 2.96–3.11), indicating that, on average, teachers’ periodontal status fell between calculus presence (CPI = 2) and shallow pocketing (CPI = 3) (Table 2) (See Figure A2).

The sample distribution was 43.0% males and 57.0% females, nearly even across the three governorates: Nablus (33.7%), Jenin (32.9%), and Tulkarm (33.4%). Most participants worked in governmental schools (72.8%), and over half reported owning their homes (52.6%). Regarding participants’ economic status, 14.2% rated their status as good/excellent, while 85.7% rated it as average/low (Table 3).

Regarding oral hygiene status, most participants (95.3%) demonstrated a moderate (S-OHI) score, while only 6.3% reported brushing their teeth two or more times daily. More than half of the participants (52.6%) reported brushing for 1 min or less, whereas fewer reported brushing for more than 1 min. The Modified Bass brushing technique was practiced by only 2.4% of the sample, and 7.0% reported using medium-bristled toothbrushes. Regarding dental care behaviours, 43.9% of participants reported attending dental visits regularly, yet only 3.9% sought care specifically for periodontal cleaning. Regarding toothbrush replacement habits, 36.9% of participants reported changing their toothbrush every 2 months, while the majority (63.1%) changed their toothbrush every 3 months or more frequently (Table 4). Overall, the most reported intake frequencies for fruits, vegetables, red meat, fish, and pastries were 2–4 times per week, with a range of 28.3% to 37.0% of participants. In contrast, daily consumption (once per day or more than 6 times per week) was less frequent for these food items, with proportions generally ranging from 7% to 21%. Nut consumption demonstrated a relatively even distribution across categories, with moderate intakes (14–22%) being more commonly reported, and daily intake ranging from 9 to 11%. Notably, sweets and soda showed higher levels of frequent consumption, with 18–19% of teachers consuming them 6 times per week and approximately 10% reporting daily intake (Table A1).

In the bivariate logistic regression analysis, several sociodemographic factors were found to be significantly associated with periodontal status. Participants from Nablus (Crude OR = 2.67) and Jenin (Crude OR = 1.61) were more likely to have PD compared with those from Tulkarm. Participants reporting good/excellent economic status had lower odds of PD compared with those reporting average/low status (Crude OR = 0.39). The number of children, the number of family members, and house ownership were also significant variables for periodontal status among the participant teachers (Table 3).

Teachers with a good oral hygiene status, as assessed by (S-OHI), had significantly higher odds of maintaining a healthy periodontal condition compared with those with poor hygiene (Crude OR = 3.70). Brushing twice or more daily was a strongly protective determinant, reducing the odds of periodontal problems by almost 86% compared with those who did not brush (Crude OR = 0.14). Similarly, teachers who brushed for one minute or less were more than twice as likely to present with poor periodontal status compared with those who brushed for two minutes or more (Crude OR = 2.04). Practising the Modified Bass technique was highly protective, showing nearly a sevenfold increase in odds of healthy periodontal status (Crude OR = 6.80). Moreover, the use of a medium-bristle toothbrush was significantly associated with better periodontal health (Crude OR = 2.77). Teachers who reported regular dental visits also had twice the odds of healthier periodontal conditions compared with those who did not (Crude OR = 2.11). Conversely, those who visited the dentist specifically for periodontal cleaning were less likely to present with a healthy periodontal status (Crude OR = 0.38) (Table 4).

Teachers’ dietary habits are associated with their periodontal health. Specifically, increased fruit consumption significantly correlates with a reduced likelihood of PD (Crude OR = 0.625). This finding suggests that higher fruit intake may contribute to better periodontal outcomes, supported by a small standard error (S.E. = 0.138) and a significant Wald statistic (11.545), affirming the reliability of this result. Additionally, higher frequencies of fish (Crude OR = 0.705), nuts (Crude OR = 0.737), and red meat (Crude OR = 0.752) consumption are similarly associated with decreased odds of PD. In contrast, a higher frequency of sweet consumption is positively associated with PD (Crude OR = 1.468) (Table 5).

Three smoking-related variables showed statistically significant associations with PD. Teachers who smoked 1–35 cigarettes per week had higher odds of PD (Crude OR = 2.27), and those who smoked 36–68 cigarettes per week showed even greater odds (Crude OR = 4.47). In addition, water pipe smoking was significantly associated with PD (Crude OR = 2.20) (Table 6).

The frequencies were on/weekdays.

The final logistic regression model demonstrated a good fit to the data. The Omnibus Test of Model Coefficients was statistically significant (χ^2^ = 47.010, df = 8, *p* < 0.001), indicating that the model with predictors performed significantly better than the null model. Model performance was further evaluated using the Hosmer–Lemeshow goodness-of-fit test, which yielded a non-significant result (χ^2^ = 3.367, df = 8, *p* = 0.909), suggesting that the model fits the data well. Additionally, the Nagelkerke R^2^ was 0.525, meaning that approximately 52.5% of the variance in periodontal health status among the participants could be explained by the final set of predictors. The −2 Log Likelihood value of 51.082 in the last step further confirms the model’s adequacy.

In the final stepwise logistic regression model, some variables were excluded because they were not statistically significant (*p* > 0.05). These included the city of residence (Jenin vs. Tulkarm), the mean of S-OHI scores, the frequency of sweet consumption, fish intake, the number of family members living with the participant, and self-reported family economic status. Their exclusion indicates that these factors did not significantly contribute to predicting periodontal health outcomes in the studied population.

On the other hand, several significant predictors of periodontal health were identified with the final multivariate regression model. Participants residing in Nablus were significantly more likely to have healthy gingiva than those from Tulkarm (AOR = 75.997).

Frequent tooth brushing was strongly associated with improved periodontal status (AOR = 0.015), as was morning brushing (AOR = 0.015). Regular replacement of toothbrushes was also a significant predictor of better periodontal health (AOR = 2.514).

Regarding dietary habits, higher consumption of red meat was significantly associated with worse periodontal outcomes (AOR = 0.032), whereas more frequent consumption of nuts appeared to be protective (AOR = 0.227).

Additionally, the number of cigarettes smoked per week was positively associated with PD (AOR = 1.085) (Table 7).

## 4. Discussion

PD is a prevalent chronic inflammatory condition that affects the supporting structures of the teeth and remains a leading cause of tooth loss among adults globally [59]. The present study assessed the prevalence of PD and examined its associations with potential determinants, including SES, oral hygiene practices, dietary habits, and smoking behaviors, among 9th-grade teachers from both governmental and private schools in the northern governorates of the West Bank. Notably, this teacher (adult) sample was drawn from the same educational setting where their 9th-grade students (adolescents) were examined in a parallel study [20].

Early detection and management of PD are critical for preventing its onset and progression and for mitigating associated systemic complications, such as cardiovascular disease, diabetes mellitus, and adverse pregnancy outcomes [60]. Identifying periodontal problems in adults is essential not only to improve their oral health-related quality of life but also to address the role adults play as role models who influence children’s oral health behaviors. Adults’ attitudes and practices toward oral hygiene strongly shape the development of healthy habits in children and adolescents [61].

A major strength of this study lies in its robust and representative sample of 920 teachers, selected using a multistage proportional stratified random sampling approach across three central governorates—Nablus, Jenin, and Tulkarm. Additionally, the study employed validated and standardized instruments for data collection, including the WHO’s Community Periodontal Index for Treatment Needs (CPITN), the Simplified Oral Hygiene Index (S-OHI), and a structured, closed-ended questionnaire. The reliability of the clinical assessments was reinforced by strong inter-examiner agreement, with intraclass correlation coefficients (ICCs) of 0.79 for CPITN and 0.89 for S-OHI, indicating high consistency and methodological rigor.

CPITN is a valuable and reliable tool for conducting epidemiological surveys [45]. It offers a more straightforward approach than methods that require detailed measurements of clinical attachment loss, such as the Basic Periodontal Examination (BPE), which is primarily suited for routine clinical screening [62]. In turn, the CDC/AAP case definitions were not applied in this study because they are mainly designed for surveillance and clinical case classification systems used in epidemiological surveillance in the United States [63]. However, the present study focused on community-based assessment using the CPITN, which emphasizes essential indicators, including gingival bleeding, the presence or absence of supra- or subgingival calculus, and periodontal pockets.

CPITN findings align well with international standards, and its findings have been included in over 500 publications. Furthermore, in larger studies, the extensive time and resources required for detailed Clinical Attachment Level (CAL) measurements can be quite considerable. Consequently, CPITN is a more cost-effective and practical alternative for periodontal assessments [64].

The findings of this study reveal an overall unfavorable periodontal health profile among the adult participants, with only a small proportion exhibiting completely healthy gingiva. The average periodontal condition among teachers ranged from the presence of calculus to shallow pocketing, indicating early to moderate disease activity. These results align with reports from South American studies in Brazil, Argentina, and Chile, which similarly documented widespread periodontal problems among adults [65]. In contrast, populations in more industrialized countries, such as the Netherlands, have shown steady improvements in periodontal health over recent decades, reflecting the impact of preventive public health measures and regular dental attendance [66]. Compared with neighboring countries, the periodontal situation in the West Bank appears less favorable; studies from Jordan have reported comparatively lower disease levels [67]. Such regional disparities likely reflect differences in oral health awareness, accessibility of preventive care, and the effectiveness of community-based oral health programs.

Following WHO recommendations for CPITN-based epidemiological surveys [44], periodontal status was assessed by mouth region to identify site-specific variations. The lower right second molar area demonstrated the healthiest gingival condition, which may relate to hand dominance and brushing-related dexterity. Because most individuals are right-handed, the left side of the mouth often receives more effective brushing, especially in posterior regions [68,69]. In addition, the proximity of the lower molars to salivary ducts and the tongue’s natural movement may aid in plaque clearance, enhancing gingival health in this area [70]. Conversely, the upper central incisor region showed the greatest tendency for gingival bleeding. This could stem from its anatomical prominence and thinner gingival tissue, which are more prone to plaque accumulation and inflammatory responses at the gingival margin during probing.

Several determinants of PD among 9th-grade teachers were retained in the final regression model. The frequency of tooth brushing emerged as the most significant predictor of periodontal health in the final regression model, with an AOR of 0.015. This finding indicated that individuals who maintain a regular toothbrushing routine are less likely to exhibit signs of PD. This finding is consistent with the existing literature, which emphasizes the importance of tooth brushing in maintaining oral health and reducing the risk of periodontal conditions [71,72]. Indeed, many patients struggle to remove plaque effectively through their home oral hygiene practices. As a result, most dental professionals recommend brushing teeth twice daily to manage plaque removal effectively [73,74]. Increasing the frequency of dental brushing can help mitigate potential shortcomings in individual brushing techniques and dexterity, especially in effectively reaching all tooth surfaces. More frequent brushing enhances the chemical removal of plaque by the active ingredients in toothpaste. This practice helps maintain a cleaner oral environment, reduces bacterial colonisation, and consequently lowers the risk of PD. Therefore, promoting regular brushing is not only beneficial for hygiene but also serves as a proactive strategy for preventing common oral diseases through both mechanical and chemical means.

Morning tooth brushing also showed a strong association with periodontal health, corroborating previous findings by Kaneyasu et al. [75]. Brushing in the morning removes the bacterial accumulation that develops overnight and neutralizes the acidic oral environment created by reduced salivary flow during sleep [ibid]. During sleep, saliva flow diminishes, creating an environment conducive to bacterial proliferation. Morning brushing addresses this accumulation before it can irritate the gingiva [76]. Moreover, morning brushing disrupts plaque formation before it hardens into calculus. Plaque that remains unaddressed may gradually mineralize into calculus, which is significantly more challenging to eliminate and substantially contributes to periodontal inflammation [77].

Dietary habits played a significant role, particularly the frequency of red-meat consumption, which was inversely associated with periodontal health. A high intake of red meat may contribute to oxidative stress and chronic inflammation through heme-iron-mediated tissue damage and the increased production of pro-inflammatory cytokines, such as IL-6 and TNF-α [78]. In addition, diets rich in saturated fats and poor in plant-based foods may alter the oral microbiota, favouring the proliferation of pathogenic species [79]. These biological mechanisms, compounded by lifestyle factors such as smoking, stress, and lower socioeconomic status, could explain the observed association [80,81,82].

The frequency of toothbrush replacement is significantly associated with periodontal health, as reflected in an AOR of 2.514. Participants who changed their toothbrushes more regularly had approximately 2.5 times the odds of maintaining better periodontal health compared to those who changed their toothbrushes less frequently. This finding is consistent with the study by Pérez-Portilla et al. [72], which found that regularly replacing toothbrushes is crucial for minimizing bacterial accumulation. Intact and properly aligned bristles provide the mechanical action necessary to disrupt and remove dental plaque biofilms effectively. Over time, bristles wear and fray, diminishing their ability to clean thoroughly and allowing bacteria to accumulate on tooth surfaces and along the gingival margins. By replacing toothbrushes, the bristles maintain their structural integrity, enhance cleaning effectiveness, and promote healthier gingival and periodontal conditions [73]. Additionally, the literature suggests that the design of toothbrush bristles and heads fosters an ideal environment for bacterial growth. Over time, bristles accumulate toothpaste residue, food particles, saliva, and moisture, which can promote bacterial proliferation. This can lead to the formation of colonies of pathogens such as Porphyromonas gingivalis. Each use of a contaminated toothbrush can reintroduce these microorganisms into the mouth. While the oral cavity naturally hosts various bacteria, reintroducing pathogenic strains can disrupt the balance of oral microbiota and potentially trigger or worsen gingival inflammation and periodontal disease [83]. Finally, worn toothbrushes can cause minor trauma or micro-abrasions to gingival tissues, increasing the risk of inflammation and infection from harmful bacteria on contaminated bristles. Therefore, regular replacement of toothbrushes is crucial to minimizing microbial contamination, reducing bacterial exposure, and improving periodontal health [84].

This study revealed a significant association between the frequency of nut consumption and periodontal health (AOR = 0.227). Participants who regularly consumed nuts had approximately a 77% lower likelihood of experiencing poor periodontal health compared to those who consumed nuts infrequently; these findings are consistent with several other studies [85,86]. The positive link between nut consumption frequency and periodontal health is attributable to the nutritional profile of nuts. Nuts are rich in anti-inflammatory compounds, antioxidants, and essential nutrients, such as omega-3 fatty acids, which help moderate the inflammatory response and reduce tissue damage caused by chronic inflammation [87]. Additionally, vitamin E neutralizes free radicals and decreases oxidative stress, a significant contributor to chronic inflammation and damage to periodontal tissues. By lowering oxidative stress, vitamin E promotes healthier periodontal tissues and reduces gingival inflammation [88]. Furthermore, magnesium supports immune function by regulating cytokine production, promoting effective immune responses, and reducing inappropriate inflammatory reactions [86]. Dietary fibers also contribute by stimulating salivary secretion and mechanically aiding in the removal of plaque and food debris during chewing [89].

The city of residence (Nablus vs. Tulkarm) was also significantly associated with periodontal health. Participants from Nablus had higher odds of exhibiting a healthier periodontium than those from Tulkarm. This notable difference suggests that geographic or environmental factors specific to each city may significantly influence oral health [88]. These factors may include variations in access to dental care services [90], differences in oral health education and awareness programs, dietary practices, and socioeconomic conditions [91].

Another key finding of this study is the strong association between cigarette consumption and PD. Each additional cigarette smoked per week was linked to a measurable increase in PD risk (AOR = 1.085), illustrating a clear dose–response relationship. This implies that even modest increases in smoking frequency can substantially elevate disease likelihood; individuals who smoked ten additional cigarettes weekly had more than twice the odds of developing PD compared with non-smokers. These results provide valuable evidence for public health initiatives aimed at reducing tobacco use by demonstrating that incremental reductions in smoking frequency can yield meaningful periodontal benefits. The findings are consistent with prior studies that have documented similar dose-dependent relationships, including those by Goel et al. [92] and Khan et al. [93], both of which confirmed significantly higher odds of PD among smokers across different populations and exposure levels.

Several limitations should be acknowledged. First, the cross-sectional design restricts the interpretation of findings to associations rather than causality, as temporal relationships between the examined determinants and PD cannot be established [94]. Second, the study sample was confined to adults residing in the northern governorates of the West Bank, which may limit the generalizability of the results to other Palestinian regions, particularly rural or socioeconomically disadvantaged areas that might exhibit different risk profiles. Third, behavioural data on oral hygiene, dietary habits, and smoking were self-reported, which may introduce recall bias and social desirability effects. Participants may have under- or over-reported certain behaviors, potentially influencing the accuracy of these measures and the observed associations.

## 5. Conclusions

This study revealed a high prevalence of PD among 9th-grade teachers in the northern West Bank, with most participants exhibiting calculus or shallow pocketing, and only a small fraction exhibiting healthy gingiva. These findings confirm that PD remains a significant public-health concern even among well-educated adults. Modifiable behaviors, including toothbrushing frequency and timing, regular toothbrush replacement, cigarette consumption, and dietary habits, strongly influence periodontal health. Differences in periodontal status across geographical areas may reflect Sociocultural variations and access to preventive dental services.

The findings underscore the need to prioritize adult-focused oral-health promotion strategies that enhance awareness, literacy, and motivation to adopt proper preventive behaviors. National efforts should integrate tobacco-cessation initiatives, improve access to preventive and counselling services, and strengthen collaboration between the health and education sectors. Implementing school-based oral health programs that target teachers as role models could help foster a culture of prevention and long-term oral health improvement within Palestinian communities.

## Figures and Tables

**Table 1:** Distribution of Periodontal Status Across Examined Mouth Regions in Teachers.

Mouth Region	Healthy N (%)	Bleeding N (%)	Calculus N (%)	Pocket < 3 mm N (%)	Pocket ≥ 3 mm N (%)
Upper right second molar	298 (32.5)	349 (38.0)	93 (10.1)	61 (6.6)	48 (5.2)
Upper right first molar	201 (21.9)	367 (40.0)	191 (20.8)	105 (11.4)	10 (1.1)
Upper central incisor	176 (19.2)	572 (62.3)	149 (16.2)	21 (2.3)	0 (0.0)
Upper left first molar	115 (12.5)	416 (45.3)	264 (28.8)	72 (7.8)	24 (2.6)
Upper left second molar	103 (11.2)	333 (36.3)	190 (20.7)	215 (23.4)	25 (2.7)
Lower left second molar	423 (46.1)	448 (48.9)	20 (2.2)	1 (0.1)	0 (0.0)
Lower left first molar	501 (54.6)	389 (42.4)	21 (2.3)	7 (0.8)	0 (0.0)
Lower left central incisor	144 (15.7)	418 (45.5)	305 (33.2)	47 (5.1)	3 (0.3)
Lower right first molar	603 (65.7)	277 (30.2)	24 (2.6)	2 (0.2)	1 (0.1)
Lower right second molar	667 (72.7)	214 (23.3)	10 (1.1)	1 (0.1)	1 (0.1)

Note. Percentages are calculated based on the total number of valid cases for each mouth region (i.e., excluding missing data), summing to 100% within each row.

**Table 2:** Distribution of (CPITN) Categories Among Teachers.

Periodontal Status	n	%
Healthy gingiva	108	11.8
Bleeding	194	21.2
Calculus	259	28.2
Pockets 3–4 mm	273	29.8
Pockets ≥ 6 mm	83	9.1
Total	917	100

Note. Overall Community Periodontal Index score = 3.03 (SE = 0.0382; 95% CI = 2.96–3.11).

**Table 3:** Bivariate Analysis of Sociodemographic Determinants of Teachers’ Periodontal Status (Crude Odds Ratios).

Variable	Categories	Count (%)	S.E.	*p*-Value	Crude OR	95% CI	
						Lower	Upper
Gender	Male	396 (43.0%)	0.205	0.079	1.433	0.959	2.143
	Female [RC]	524 (57.0%)					
Governorate	Nablus	310 (33.7%)	0.268	**0.000**	2.67	1.577	4.518
	Jenin	303 (32.9%)	0.237	**0.044**	1.613	1.013	2.568
	Tulkarm [RC]	307 (33.4%)					
School type	Governmental	670 (72.8%)	0.222	0.276	1.274	0.824	1.968
	Private [RC]	249 (27.1%)					
No. of children	≤3	380 (41.3%)	0.250	**0.005**	0.499	0.306	0.813
	>3 [RC]	255 (27.7%					
No. of family members	≤3	55 (6.0%)	0.607	**0.000**	14.125	4.301	46.387
	>3 [RC]	230 (25.0%)					
House Ownership	Yes	484 (52.6%)	0.217	**0.001**	2.035	1.329	3.115
	No [RC]	436 (47.4%)					
Family’s economic status	Good or excellent	131 (14.2%)	0.242	**0.000**	0.394	0.245	0.632
	Average or low [RC])	785 (85.7%)					

Note. RC = Reference category; Crude OR = Crude Odds Ratio; S.E. = Standard Error; Reference group for outcome: Healthy Gingiva. No. of children (if the participant is married). No. of family members (if the participant is unmarried).

**Table 4:** Bivariate Analysis of Oral Hygiene Determinants of Teachers’ Periodontal Status (Crude Odds Ratios).

Variable	Categories	Count (%)	S.E.	*p*-Value	Crude OR	95% CI	
						Lower	Upper
S-OHI	Good	20 (2.2%)	0.266	**0.00**	3.697	2.197	6.221
	Medium	865 (95.3%)					
	Bad [RC]	23 (2.5%)					
Frequency of brushing	Once/day	493 (53.6%)	0.309	**0.052**	0.549	0.300	1.006
	Twice or more/day	58 (6.3%)	0.390	**0.000**	0.142	0.066	0.304
	Occasionally	180 (19.6%)	0.428	0.464	1.368	0.591	3.164
	Do not brush [RC]	188 (20.5%)					
Morning brushing	Yes	613 (69.8%)	0.264	**0.056**	0.604	0.36	1.013
	No [RC]	265 (30.2%)					
Duration of brushing	1 or less min.	484 (52.6%)	0.217	**0.001**	2.035	1.329	3.115
	Two or more min. [RC]	436 (47.4%)					
Use the Modified Bass technique	Yes	22 (2.4%)	0.441	**0.00**	6.803	2.864	16.157
	No [RC]	898 (97.6%)					
Use a medium-bristle toothbrush	Yes	64 (7.0%)	0.309	**0.001**	2.768	1.51	5.073
	No [RC]	856 (93.0%)					
Frequency of toothbrush replacement/months	two	267 (36.9%)	0.230	0.380	1.223	0.780	1.918
	three or more [RC]	456 (63.1%)					
Visit the dentist regularly	Yes	404 (43.9%)	0.209	**0.00**	2.109	1.4	3.177
	No [RC]	516 (56.1%)					
Reason for dentist visit	Periodontal cleaning	36 (3.9%)	0.399	**0.015**	0.379	0.173	0.829
	Other [RC]	883 (96.1%)					

Note. RC = Reference category; Crude OR = Crude Odds Ratio; S.E. = Standard Error.

**Table 5:** Bivariate Analysis of Dietary Habits as Determinants of Teachers’ Periodontal Status (Crude Odds Ratios).

Variable	S.E.	Wald	*p* Value	Crude OR	95% CI	
					Lower	Upper
Frequency of fruit-eating	0.138	11.545	**0.001**	0.625	0.477	0.82
Frequency of vegetable eating	0.138	3.502	**0.061**	0.772	0.589	1.012
Frequency of red meat-eating	0.136	4.421	**0.035**	0.752	0.577	0.981
Frequency of fish-eating	0.105	11.096	**0.001**	0.705	0.574	0.866
Frequency of nut-eating	0.103	8.784	**0.003**	0.737	0.603	0.902
Frequency of sweet eating	0.154	6.195	**0.013**	1.468	1.085	1.986
Frequency of soda consumption	0.143	2.449	0.118	1.251	0.945	1.655
Frequency of eating pastries	0.165	0.228	0.633	0.924	0.669	1.277

Note. Crude OR = Crude Odds Ratio. Reference group for outcome: Healthy gingiva.

**Table 6:** Bivariate Analysis of Smoking Determinants of Teachers’ Periodontal Status (Crude Odds Ratios).

Variable	Categories	Count (%)	S.E.	*p*-Value	Crude OR	95% CI	
						Lower	Upper
Tobacco smoking status	Yes	363 (39.5%)	0.207	0.26	1.262	0.842	1.892
	No [RC]	557 (60.5%)					
Frequency of tobacco smoking	Regularly	278 (30.2%)	0.219	0.598	1.123	0.73	1.726
	Rarely [RC]	642 (69.8%)					
Age of starting smoking(years)	10–20	332 (36.1%)	0.753	0.382	1.930	0.441	8.442
	21–30 [RC]	26 (7.3%)					
No. of cigarettes smoked/week	1–35	211 (22.9%)	0.329	**0.013**	2.273	1.192	4.334
	36–68	49 (5.3%)	0.645	**0.020**	4.472	1.262	15.846
	69 and more [RC]	93 (10.1%)					
Smoking status (water pipe)	Yes	811 (88.2%)	0.272	**0.024**	2.2	1.3	3.7
	No [RC]	109 (11.8%)					
Frequency of water pipe smoking	Frequently	721 (78.4%)	0.283	0.16	0.672	0.386	1.17
	Rarely [RC]	127 (13.8%)					

**Table 7:** Final Multivariate Logistic Regression Model for Predictors of Periodontal Health.

Predictors	S.E.	Wald	*p*-Value	Adjusted OR	95% CI	
					Lower	Upper
City (Nablus vs. Tulkarm)	1.900	5.193	0.023	75.997	1.833	3150.804
Frequency of brushing	1.009	17.551	0.000	0.015	0.002	0.105
Brushing in the morning	1.353	9.784	0.002	0.015	0.001	0.206
Frequency of toothbrush changing	0.354	6.797	0.009	2.514	1.257	5.026
Frequency of red meat eating	1.278	7.210	0.007	0.032	0.003	0.396
Frequency of nut eating	0.619	5.728	0.017	0.227	0.067	0.765
Number of cigarettes smoked per week	0.040	4.094	0.043	1.085	1.003	1.173

Note. Adjusted OR = Adjusted Odds Ratio. Reference group for outcome: Healthy gingiva.

## Data Availability

The datasets analysed during the current study are available from the corresponding author upon reasonable request. Controlled access is required due to institutional and Ministry of Education policies, which necessitate a data-use agreement and review of the research purpose. Requests may be directed to the corresponding author at [sura.kazlak@yahoo.com] and will be considered in line with the approved protocol (REC approval: REF.13/24).

## Abbreviations

The following abbreviations are used in this manuscript:

Abbreviation	Meaning
AOR	Adjusted Odds Ratio
BPE	Basic Periodontal Examination
CAL	Clinical Attachment Level
CI	Confidence Interval
CPITN	Community Periodontal Index for Treatment Needs
df	Degrees of Freedom
EFA	Exploratory Factor Analysis
EPV	Events Per Variable
ICC	Intraclass Correlation Coefficient
KMO	Kaiser–Meyer–Olkin (measure of sampling adequacy)
K-NN	K-Nearest Neighbour (missing-data imputation)
S-OHI	Simplified Oral Hygiene Index
OR	Odds Ratio
PCA	Principal Component Analysis
RC	Reference Category
SE	Standard Error
SES	Socioeconomic Status
SPSS	Statistical Package for the Social Sciences
WHO	World Health Organisation

## Appendix A

**Table A1:** Description of Dietary Habits of the Participant Teachers.

No.	Description	Code/Values	Count (% of Total)
1	Frequency of fruit-eating	Never	70 (7.6%)
		Less than once/week	120 (13.0%)
		Once/week	150 (16.3%)
		2–4 times/week	265 (28.8%)
		6 times/week	190 (20.7%)
		Once/day	70 (7.6%)
		More than once/day	55 (6.0%)
2	Frequency of vegetable-eating	Never	65 (7.1%)
		Less than once/week	90 (9.8%)
		Once/week	130 (14.1%)
		2–4 times/week	340 (37.0%)
		6 times/week	160 (17.4%)
		Once/day	80 (8.7%)
		More than once/day	55 (6.0%)
3	Frequency of red meat-eating	Never	80 (8.7%)
		Less than once/week	100 (10.9%)
		Once/week	140 (15.2%)
		2–4 times/week	295 (32.1%)
		6 times/week	160 (17.4%)
		Once/day	90 (9.8%)
		More than once/day	55 (6.0%)
4	Frequency of fish-eating	Never	90 (9.8%)
		Less than once/week	110 (12.0%)
		Once/week	120 (13.1%)
		2–4 times/week	260 (28.3%)
		6 times/week	180 (19.6%)
		Once/day	90 (9.8%)
		More than once/day	68 (7.4%)
5	Frequency of nut eating	Never	100 (10.9%)
		Less than once/week	130 (14.1%)
		Once/week	160 (17.4%)
		2–4 times/week	200 (21.7%)
		6 times/week	150 (16.3%)
		Once/day	100 (10.9%)
		More than once/day	80 (8.7%)
6	Frequency of sweet eating	Never	60 (6.5%)
		Less than once/week	110 (12.0%)
		Once/week	140 (15.2%)
		2–4 times/week	279 (30.4%)
		6 times/week	170 (18.5%)
		Once/day	90 (9.8%)
		More than once/day	70 (7.6%)
7	Frequency of soda consumption	Never	75 (8.2%)
		Less than once/week	95 (10.4%)
		Once/week	150 (16.4%)
		2–4 times/week	260 (28.4%)
		6 times/week	160 (17.4%)
		Once/day	100 (10.9%)
		More than once/day	55 (6.0%)
8	Frequency of eating pastries	Never	85 (9.2%)
		Less than once/week	120 (13.0%)
		Once/week	135 (14.7%)
		2–4 times/week	295 (32.1%)
		6 times/week	140 (15.2%)
		Once/day	90 (9.8%)
		More than once/day	77 (8.4%)
